# Incidence of Infection Following Local Anesthetic Transperineal Prostate Biopsy: A Single-Centre Experience

**DOI:** 10.7759/cureus.61907

**Published:** 2024-06-07

**Authors:** Baidar Khalabazyane, Christine Mizzi, Rahel Rashid, Lawrence Adesunloro, Roza Salah, Israa Kadhmawi, Priyadarshi Kumar

**Affiliations:** 1 Urology, Royal Bournemouth Hospital, Bournemouth, GBR; 2 Urology, University Hospitals of Coventry and Warwickshire, Coventry, GBR; 3 General and Colorectal Surgery, Arrowe Park Hospital, Wirral, GBR; 4 Plastic and Reconstructive Surgery, Salisbury Foundation Trust, Bournemouth, GBR; 5 Medicine, Arrowe Park Hospital, Wirral, GBR

**Keywords:** post-latp infection, transrectal prostate biopsy, risk factors of infection, antibiotic prophylaxis, prostate biopsy, transperineal prostate biopsy, latp

## Abstract

Background

Local anesthetic transperineal prostate biopsy (LATP) is a widely used diagnostic procedure for prostate cancer. As a diagnostic procedure, it should carry minimal risk. However, morbidity resulting from prostate biopsy is frequent. Prostate biopsy, like any other intervention, carries a significant risk of various infections, ranging from urinary tract infections (UTIs) to potentially life-threatening conditions like sepsis.

Aim

This study examined the rate of infections following a prostate biopsy at a single center and sought to identify risk factors that could increase the likelihood of developing an infection.

Methods

A retrospective review was conducted on all 168 patients who underwent LATP biopsy between 01/04/2022 and 01/04/2023. Data were collected from the Clinical Record and Reporting System (CRRS). Patient characteristics, including age, prostate-specific antigen (PSA) levels, prostate volume, the main indication for the biopsy, number of cores taken, antibiotic prophylaxis, and comorbidities were analyzed. The inclusion criteria encompassed all patients receiving this procedure within the specified timeframe, without restrictions on age, underlying health conditions, or medical history. No exclusion criteria were applied, aiming to comprehensively analyze and capture the full spectrum of patient outcomes and characteristics associated with these biopsies during the study period.

Results

In terms of socio-demographics, all patients were male with an average age (mean) of 65.5 years, a mean PSA level of 13.9 ng/dL, and an average prostate volume of 66.1 mL. On average, 23.2 biopsy cores were taken. All patients received antibiotic prophylaxis, mainly ciprofloxacin. Despite this, 1.78% of patients (n=3) developed post-biopsy infections. Two of these patients had diabetes mellitus, and two had a large prostate volume of 95 mL.

## Introduction

Prostate cancer is one of the most common malignancies affecting men worldwide, ranking second in diagnosis frequency and fifth in global mortality. Timely detection and accurate diagnosis are imperative for optimizing treatment and enhancing patient outcomes [1].

Transrectal (TR) prostate biopsy remains the most commonly used diagnostic tool for prostate cancer in most regions worldwide [2]. It has an infection risk ranging from 5% to 7% [3].

Local anesthetic transperineal prostate biopsy (LATP) is a widely accepted and well-tolerated procedure for diagnosing prostate cancer. It offers several advantages over traditional TR biopsy, including reduced infection and sepsis risk, improved sampling of the apical and anterior prostate regions, and potentially higher cancer detection rates [4-6].

Among men under active surveillance for low-risk prostate cancer, LATP is more adept at identifying clinically significant cases, likely due to improved sampling of the anterior prostate region [7].

A systematic review found that using antibiotic prophylaxis for LATP did not significantly reduce the rates of infection, fever, sepsis, or hospital readmission compared to cases without antibiotic prophylaxis [8]. However, despite its benefits, LATP is an invasive procedure that inevitably carries some risk of post-biopsy infection which can range from uncomplicated urinary tract infections (UTIs) to severe sepsis, potentially leading to hospitalizations and increased healthcare costs [9].

Recognizing risk factors for post-biopsy infections is vital for prevention and enhancing patient care. This study evaluated the post-biopsy infection rate following LATP at a single center and sought to identify potential risk factors contributing to infection.

## Materials and methods

This was a retrospective, single-center study conducted at the University Hospitals of Coventry and Warwickshire in the United Kingdom. Data were collected from the Clinical Record and Reporting System (CRRS) for all patients who underwent LATP biopsy from 01/04/2022 to 01/04/2023. A total of 168 patients were included in the study.

The collected data included patient characteristics like age, prostate-specific antigen (PSA) levels, prostate volume, main indication for the biopsy, number of biopsy cores, antibiotic prophylaxis use, and co-morbidities linked to immunosuppression or increased infection risk (e.g., diabetes mellitus, hematologic disorders, and self-catheterization). Microsoft Excel was used to calculate the average (mean), standard deviations (SD), and any other statistical analyses carried out.

## Results

The study included 168 patients who underwent LATP biopsy. The average (mean) age of the patients was 65.5 years (SD 8.E+00). The mean PSA level was 13.9 ng/dL (SD 3.E+01), and the average prostate volume was 66.1 mL (3.E+01). The average number of core biopsies taken was 23.2 (4.E+00). All patients received antibiotic prophylaxis, with ciprofloxacin being the most used (167 patients), followed by gentamicin (one patient only).

**Figure 1:**
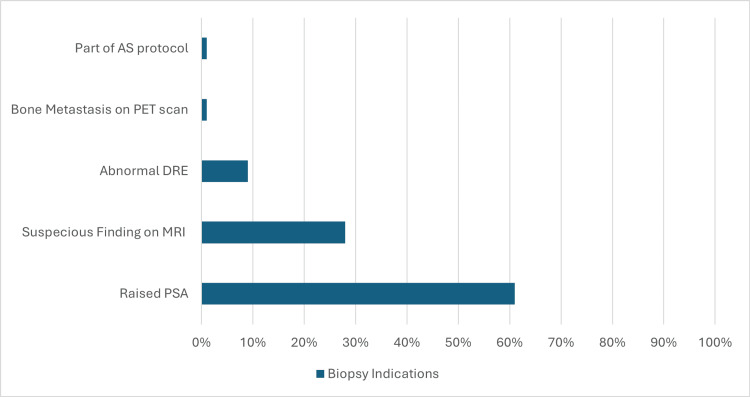
Main indications for biopsy AS: active surveillance, PET: positron emission tomography, DRE: digital rectal examination, MRI: magnetic resonance imaging, PSA: prostatic specific antigen

The main indications for biopsy were raised PSA (103 patients), suspicious findings on MRI (48 patients), abnormal digital rectal examination (DRE) (15 patients), and bone metastasis on positron emission tomography (PET) scan (one patient), as part of active surveillance protocol (one patient) (Figure 1).

A subset of patients had comorbidities linked to immunosuppression or increased infection risk: 11 patients had diabetes mellitus, two had hematological disorders, and one was on intermittent self-catheterization (ISC). None had documented UTI before the procedure. Out of 168 patients, three (1.78%) developed post-biopsy infections. Two of these patients had diabetes mellitus, and two had a large prostate volume of 95 mL. None required ICU admission or additional procedures. One patient required four days of hospitalization (Table 1).

**Table 1:** Details of the patients with post-biopsy infections

Case	Date of Biopsy	Date of Infection	Urine Culture	Blood Culture	Hospitalization	Treatment	Additional Procedure	Risk Factors	Antibiotic Prophylaxis	Number of Cores
1	20/2/23	21/2/23	Negative	Not taken	4 days	10 days of co-amoxiclav	No	Large prostate volume (95 mL)	Ciprofloxacin	18
2	13/2/23	30/2/23	Negative	Not taken	Not required	Ofloxacin for 14 days	No	Diabetes mellitus, large prostate volume (95 mL)	Ciprofloxacin	18
3	4/4/23	14/4/23	Not taken	Not taken	No required	Nitrofurantoin for 7 days	No	Diabetes mellitus, moderately enlarged prostate (50 mL)	Ciprofloxacin	24

## Discussion

Prostate biopsy is a crucial procedure for acquiring tissue samples for histopathological assessment. Historically, clinicians have relied on PSA levels and DRE findings to determine the necessity of performing a prostate biopsy [10,11].

The preferred method for obtaining a prostate tissue sample has traditionally been the TR approach under ultrasound guidance. Initially, the sextant technique was regarded as the gold standard [12]. This approach systematically but randomly sampled six cores from the prostate. However, the literature highlights a high false-negative rate for this procedure, reaching up to 30% [13]. The use of quinolone-class antibiotics, typically ciprofloxacin, was recommended as prophylaxis [14]. Quinolone-class antibiotics, such as ciprofloxacin, were recommended due to their strong bactericidal activity against gram-negative bacteria (e.g., *Escherichia coli*), which are a heightened risk with TR biopsy, and their excellent penetration into prostate tissue. Additionally, there was a weak recommendation for a self-administered cleansing enema on the morning of the procedure [15]. 

Efforts to address issues like false-negative biopsies, over-sampling, and under-sampling have led to the adoption of MRI-guided biopsies. However, the TR approach is inadequate for accessing the apical and anterior regions of the prostate. Consequently, the transperineal (TP) biopsy has emerged as an alternative to overcome the limitations of the TR biopsy [16].

Though numerous studies investigate infection risk factors in TR prostate biopsy, research on risk factors of infection in LATP is sparse, likely due to procedural similarities. However, further dedicated studies are warranted to comprehensively explore this specific area.

The first step for prevention is a preoperative assessment of risk factors which includes diabetes, significant comorbidities, immunosuppression, urinary tract infections, prostatitis, the higher cumulative number of biopsies, large prostate volume, recent antibiotic use, recent international travel to resistant-endemic areas, antibiotic use for traveler’s diarrhea prevention, healthcare worker status, and colonization with resistant bacteria like fluoroquinolone-resistant *E. coli *[3].

Proper infection control measures, including equipment sterilization, using sterile ultrasound gel, avoiding contamination of tissue samples, and correct ultrasound transducer processing, are essential to prevent infectious complications after prostate biopsy [3].

Men with lower urinary tract symptoms should have a urine culture done, any infections treated, and a repeat culture performed before undergoing a prostate biopsy [3].

Interestingly, two out of the three patients who developed post-biopsy infections had diabetes mellitus. Diabetes mellitus is known to be associated with an increased risk of infections due to impaired immune function and other factors [17].

This finding suggests that patients with diabetes may be at a higher risk for post-biopsy infections following LATP and may benefit from additional preventive measures and closer monitoring.

Another notable observation was the presence of a large prostate volume in two of the cases with post-biopsy infections. It is possible that larger prostate volumes may increase the risk of complications, including infection [18].

It is important to note that all three cases of post-biopsy infection were managed with appropriate antibiotic treatment, and none of the patients required additional procedures or intensive care unit (ICU) admission. 

Potential limitations of our study include its single-center design, which may limit the generalizability of the findings to other healthcare settings or populations. Additionally, the small sample size of 168 patients may restrict the statistical power to detect significant associations or risk factors. The retrospective design, relying on data from medical records, is subject to potential errors or incomplete documentation. Furthermore, the study did not account for the duration of follow-up after the biopsy procedure, which may have led to missed or undetected infections that developed later.

## Conclusions

At this center, the post-biopsy infection rate was 1.78%, with diabetes mellitus and large prostate volume identified as potential risk factors. Larger studies are necessary to confirm these findings, identify additional risk factors and high-risk patient groups undergoing LATP, and establish necessary precautionary measures.
